# Grading bias and young adult mental health

**DOI:** 10.1002/hec.4639

**Published:** 2022-12-07

**Authors:** Anna Linder, Martin Nordin, Ulf‐G. Gerdtham, Gawain Heckley

**Affiliations:** ^1^ Health Economics Unit Department of Clinical Science Lund University Malmö Sweden; ^2^ Centre for Economic Demography Lund University Lund Sweden; ^3^ Department of Economics Lund University Lund Sweden

**Keywords:** grade inflation, grading bias, human capital development, mental health

## Abstract

We study exposure to grading bias and provide novel evidence of its impact on mental health. Grading bias, which we interpret as over‐grading, is constructed as the residual of final upper secondary school grades having controlled for results in a standardized test, itself not subject to grading leniency. Grading bias is further isolated by considering only within‐school variation in over‐grading and controlling for prior grades and school production. Using Swedish individual‐level register data for individuals graduating from upper secondary school in the years 2001–2004, we show that over‐grading has substantial significant protective impacts on the mental health of young adults, but only among female students. That grades themselves, independent of knowledge, substantially impact the production of health highlights an important health production mechanism, and implies that any changes to the design of grading systems must consider these wider health implications.

## 1 INTRODUCTION

How we grade students is hugely important because grades are used as key indicators of student performance and function as a sorting mechanism within the education system and the labor market. Systemic grading bias, over‐ or under‐grading not reflecting the true level of knowledge, could create unfairness between students. For example, if grades are inflated for the benefit of students in grading lenient schools, this will be at the expense of other, higher‐achieving students not exposed to over‐grading. Grading bias may also lead to welfare costs due to inefficient allocations of higher education investments. Various grading reforms and trends of more lenient grading have contributed to grade inflation in many countries, for example, in the U.S (Rojstaczer & Healy, 2012), Germany (Müller‐Benedict & Gaens, 2020) and Sweden (Vlachos, 2011; Wikström & Wikström, 2005). As a result, consequences of systematic grading biases have received growing attention in the literature.

Earlier studies have found that over‐grading increases higher education enrollment and achievements (Dee et al., 2019; Diamond & Persson, 2016; Maurin & McNally, 2008; Nordin et al., 2019). Over‐grading also impacts labor income, an effect that has been explained by easier access to later stages of the education system (Maurin & McNally, 2008; Nordin et al., 2019). Human capital development theory suggests that grades could impact both the production of skills and the production of health (Cunha & Heckman, 2007; Heckman, 2012). This means that health could be an outcome of grading bias that is important in itself, but health could also be a mechanism in the relationship between grading bias and labor market outcomes. Yet, to the best of our knowledge, there is no existing evidence on the potential health consequences of grading bias. Therefore, in this study, we investigate the impact of grading bias on health. Specifically, we investigate the impact on mental ill‐health among young adults which is, around the world, seen as one of the largest public health challenges in modern times (United Nations Children's Fund, 2021). We hypothesize that over‐grading impacts the production of mental health through (1) a direct effect of performance feedback, (2) an impact on self‐efficacy beliefs by which positive feedback/re‐ranking increases self‐confidence, motivation and performance thereby positively affecting mental health, or (3) through the admission distortion which increases opportunities for higher education investments, of which both attendance and years completed have been found to causally affect health (Buckles et al., 2016; Heckley et al., 2022).

Several reforms related to deregulation and decentralization of the Swedish school system were implemented at the beginning of the 1990s including a major reform of the grading system. The reforms introduced competition in the Swedish school market which, in combination with a move from a relative to a goal‐ and criterion‐referenced grading system, contributed to substantial grade inflation during the following decade (Vlachos, 2011; Wikström & Wikström, 2005). Our data includes all students who graduated from upper secondary schools in Sweden between the years 2001–2004, that is, students exposed to grade inflation following the 1990s school reforms. For these cohorts, one in eight students had a psychiatric‐related diagnosis or prescription in the years after graduation, which is comparable to more recent, as well as international, prevalence (United Nations Children's Fund, 2021). To identify grading bias at the school level we exploit the difference between upper secondary Grade Point Average (GPA) and standardized Swedish Scholastic Aptitude Test (SweSAT) results, a test not subject to grading leniency. On an individual level, the difference between grades and SweSAT results is a residual skill measure, reflecting grading error and variation in non‐cognitive skills. However, on the school level, the difference between upper secondary school grades and SweSAT results capture systemic changes in grades that can be used to identify grading bias, at least if we control for non‐cognitive skills.

Within‐school changes in grading bias, controlling for prior cohort knowledge, background characteristics and changes in school production, finds that being exposed to over‐grading has a significant, protective, impact on mental health, but only among women. The changes in grading bias are assumed to reflect increased levels of over‐grading since they are measured over a period when grade inflation was observed. A quarter of a standard deviation increase in over‐grading, exhibited by more than a third of schools in our sample, is associated with an eight percent lower probability of being diagnosed for internalizing disorders such as depression and anxiety, and a four percent lower probability of being prescribed mental ill‐health related drugs such as antidepressants and anxiolytics. The effect appears a couple of years after graduation, which suggests that it is more likely to be a self‐efficacy effect than a direct effect of performance feedback. It also appears as though this effect is driven by women with low SES fathers and by women with no migration background.

These findings are important for several reasons. First, we show that the consequences of systematic grading bias are complex and go beyond the schooling and labor market consequences previously suggested in the literature—over‐grading also impacts mental health—at least for women, which means that grades themselves, independent of knowledge changes, are important for both health and skills formation among students. Secondly, this has important policy implications. Given that grading bias has a non‐negligible impact on mental health, any policy that directly or indirectly impacts grading in schools, for example, blind/non‐blind grading of tests and the choice between standardized or discretionary grading systems, may also impact health and life chances among these young individuals, potentially in unintended ways. Future policy should consider this and be careful when manipulating factors that have implications for student grades.

The paper is organized as follows: in the next section, we present a conceptual framework where we discuss the relationship between grading bias and mental health in the context of the theory of developmental origins. In section three, we give a brief background to the Swedish schooling system with regards to the grading system and the relevant school reforms that impacted grade inflation. Sections four and five present our data and empirical strategy. In section six, we present the main results, as well as an extended investigation of potential timing, heterogeneity and cause‐specific effects. In section seven, we provide results of a number of robustness tests that provide support of our chosen identification strategy. Finally, in sections number eight and nine, we discuss and conclude our findings.

## 2 CONCEPTUAL FRAMEWORK

To help conceptualize how systematic grading bias could impact the development of mental health in young adult life, we turn to the human capital development literature in economics, an investment framework that unifies the literature on skills formation and production of health, see Cunha and Heckman (2007) and Heckman (2012). This framework describes how future outcomes such as education or earnings depends on one's current stock of capabilities. Capabilities at any given time consist of cognitive and non‐cognitive skills (e.g., motivation, time preferences and social competence), and health. The idea is that the stock of capabilities evolves and that higher skills or better health in one period can affect the returns to any investment and thereby the stock of these capabilities in the next period (Cunha & Heckman, 2007). They are also potentially cross‐fertilizing, for example, that emotional stability, motivation and good health can support the learning process, or that self‐regulation and conscientiousness can reduce health risks (Cunha & Heckman, 2007). School grades reflect each student's cognitive and non‐cognitive skills, but grades may also include systematic grading bias, that is, over‐ or under‐grading. If grading bias has a direct impact on health or skills, or an impact on the possibility to make investments in health or skills, the potential impacts of school‐level grading bias may be observed across the full vector of capabilities, including mental health.

There are several potential causal channels for grading bias to influence the stock of capabilities, including mental health. First, there may be a direct link between over‐grading and mental health. Grades are a form of feedback and feedback has been linked to several dimensions of mental health (Gustafsson et al., 2010). This is based on theories of symbolic interactionism, that children's perceptions about themselves are based on other people's appraisals about them, and that frequent positive (or negative) feedback impacts children's self‐image which inhibits (or promotes) depression (ibid.). This would mean that being exposed to over‐grading would have an immediate protective effect on an individual's stock of capabilities, through its impact on mental health.

A second potential channel is through the impact of over‐grading on self‐efficacy beliefs. A central question in social cognitive theory is related to how self‐efficacy beliefs regulate cognitive, motivational, affective, and decisional processes (Bandura & Locke, 2003). Such beliefs could, for example, affect the choices we make at important decisional stages and if we think and act in self‐enhancing or self‐debilitating ways. They could also impact how we motivate ourselves in demanding situations and our vulnerability to stress and depression. According to Bandura and Locke (2003), self‐efficacy beliefs are linked to differences in motivation and performances between individuals, but they are also linked to such differences in the same person over time. This means that self‐efficacy beliefs could be responsive to changes in feedback that is a result of grading bias and, in turn, have an impact on mental health. Any change in self‐efficacy would result in a direct change in the stock of capabilities, but if self‐efficacy only impacts mental health through later positive feedback that is a consequence of the increased self‐efficacy, this may take a while to impact the stock of mental health, in which case we would find the impact of grading bias on mental health only after a couple of years.

Third, over‐grading could impact mental health through increased higher education enrollment opportunities. It has been shown that over‐grading increases university enrollment. Schooling is linked to better mental health, but schooling is also expected to have positive impacts on later labor market outcomes, which also are correlated with better mental health (Lund et al., 2018; Macintyre et al., 2018). Indirect effects of over‐grading on mental health, through schooling and labor market outcomes, would likely be revealed much later in life.

Another aspect of the human capital development framework is that an individual's background characteristics, for example, social environment, migration background, parents' education and income, can be important for the development of capabilities. This is because acquired skills, health and investments over different stages of childhood bolster each other, and children from lower resource families usually have fewer opportunities to benefit from such multiplier effects which, according to (Cunha & Heckman, 2007), is a potential contributor to the socioeconomic gradient in health. It is possible that the three paths we hypothesize as potential channels between grading bias and mental health vary with family background. They may also vary based on gender. If constraints to higher education differ between groups in the population, or if self‐efficacy responses to grades manifest themselves differently based on background characteristics and between women and men, then grading biases may also contribute to health gaps in the population. Previous findings indicate that this could be the case. Lavy and Sand (2015) show that teachers' discretionary grading favoring boys in math and science has a lasting positive effect on achievements and enrollment in advanced level courses among boys, yet a negative effect on girls. They also show that grading bias has spill‐over effects across subjects (Lavy & Sand, 2015). Terrier (2015) concludes the same but for the opposite sex, when girls are over‐graded in comparison to boys, they progress more, which indicates that grades themselves impact the development of skills also among women. Lavy and Sand (2015) moreover showed that the negative and positive impacts of grading bias on later school performance were intensified by both a socioeconomic and an ethnic gradient. Maurin and McNally (2008) showed that grading bias increased higher education enrollment among students on the margin of passing secondary school, reducing education gaps (since those on the margin of passing were more likely to be low socioeconomic status). Nordin et al. (2019) showed that over‐grading increased university enrollment among men but not among women, and moreover that low‐skilled women were affected negatively, potentially suggesting a negative self‐efficacy effect of not being exposed to over‐grading on school achievements among women.

## 3 THE SWEDISH SCHOOLING SYSTEM

Several large reforms were implemented in the Swedish school system at the beginning of the 1990s. One reform, enacted in 1992, was related to a decentralization of Swedish schools which meant that the responsibility for primary and secondary schools was shifted from the central state to the local municipalities. Another reform introduced in the same year involved deregulation of the school financing which meant that privately run schools could now receive public funding through a voucher system. A third reform was related to school choice and increased the possibility for students to choose schools outside of their catchment area. Moreover, in 1994, the grading system was reformed from a norm‐referenced relative system to a criterion‐based goal‐oriented system. In the prior system, grades were based on a scale from 1–5 and on a national level, the average grade should be a 3 with a standard deviation of 1. National standardized tests were given to help teachers grade the students according to the norm distribution, the class mean GPA should not diverge from the class mean of the national tests. With the reform, grades were based on the scale *fail* (IG), *pass* (G), *pass with distinction* (VG) and *pass with special distinction* (MVG). The final grade was calculated such that the points for each course grade (10 points for G, 15 points for VG, and 20 points for MVG) were weighted by the credits for each course. Thus, each student's final grade lied between 0 and 20, and the distribution of grades was now based on how well the student met specific learning criteria in each subject, instead of a relative knowledge/performance comparison to one's peers. National course tests were also used in some subjects after the reform to guide the teachers in the grading process but the responsibility for grading is completely decentralized to the schools and the teachers, which means that the transparency of the grading in schools is quite low.

In Sweden, both upper secondary GPA and the SweSAT results function as ranking instruments for admission to higher education. The SweSAT is taken outside of schools and is administered by the Swedish Council for Higher Education. The test is offered twice a year, and the difficulty level may vary between the occasions, which is why the test score is normalized on a scale from 0.0 to 2.0. Around one third of students are admitted through the SweSAT. Since upper secondary grades are based on the teacher's comprehensive assessment of student performance in relation to goals and grading criteria for each course, generally, grades reflect both cognitive and non‐cognitive skills (e.g., presentation skills, ability to collaborate). Contrarily, since the SweSAT score is not subject to teacher discretion, the results are less likely to be biased.

**FIGURE 1:**
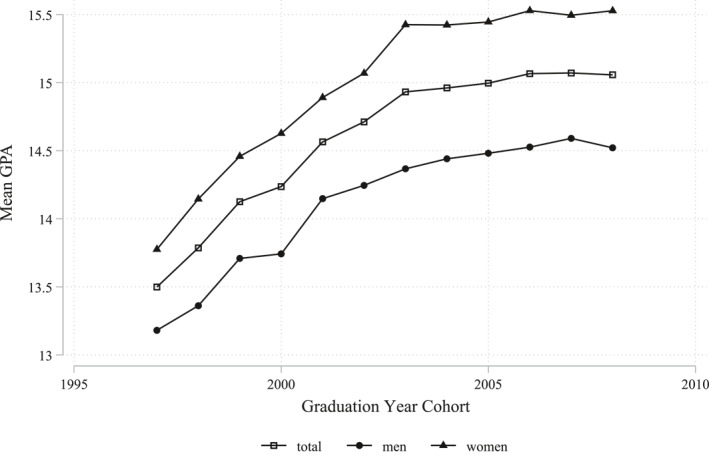
Trends of mean upper secondary grades. This figure shows the trends in mean grade point average (GPA) among students graduating from academic track in upper secondary schools in Sweden in the years 1997–2008 (see Section 4 for specific information about the sample). The grading scale runs from 0 to 20.

The deregulation and the marketization of the Swedish school system at the beginning of the 1990s introduced competition between schools. Because of the increased inter‐school pressures, in combination with the restructuring of the Swedish grading system in 1994, teachers now had both the incentive and the possibility to over‐grade, which resulted in substantial grade inflation in upper secondary schools in Sweden during the following decade (Cliffordson, 2004; Vlachos, 2011; Wikström & Wikström, 2005). As shown in Figure 1, mean upper secondary GPA increased markedly both among students graduating from academic track in upper secondary schools after the introduction of the goal‐oriented grading system, with the first cohort graduating in 1997. The general grade inflation trend reduced after 2003, although a weaker increase in grades can be found among men until 2007, and for some years also among women. This trend break is likely related to the growing awareness of grade inflation during the first years of the 2000s which resulted in several reports highlighting the shortcomings and lack of equality with the new grading system, and also culminated in an audit by The Swedish National Audit Office in 2004.

**FIGURE 2:**
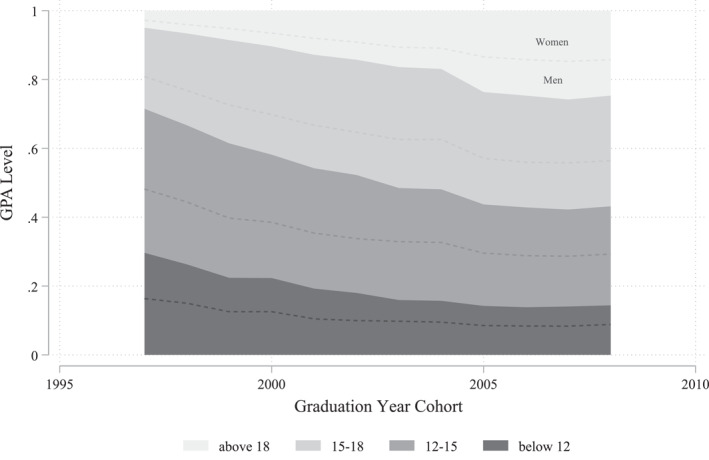
Trends in the distribution of different grade levels. This figure shows the trends for the distribution of different grade point average levels (0–11.99, 12–14.99, 15–17.99, and 18–20) among students graduating from academic track in upper secondary schools in Sweden in the years 1997–2008 (see Section 4 for specific information about the sample). For each grade point average (GPA) level, the share of women (men) is shown above (below) the dashed line. The grading scale runs from 0 to 20.

Figure 2 shows the relative distribution of GPA levels divided in four different levels. As shown in the figure, students in the two highest grade levels increased significantly between 1997 and 2005. The top level share grew five times from roughly 5% to 24% and the second highest grade level grew by one‐third from 24% to 33%. The increase can be seen both among women and men, but is especially strong among women. In the following years the distribution remained roughly stable, but a slightly increasing trend was found in the top grade level group.

## 4 DATA

We use a population sample of students graduating from upper secondary schools in Sweden in the years 2001–2004 (*n* = 299,459). To make sure that the grading bias measure is reliable we exclude students in schools that have less than 240 students in the four cohorts, that is, less than on average 60 students per cohort (*n* = 26,126 excluded). Only around 15% of students on the vocational tracks take the SweSAT, therefore, the sample is also restricted to students graduating from an academic track in upper secondary school, resulting in a substantial drop in our working sample (*n* = 136,915 excluded). Finally, since we need compulsory school GPA to control for non‐cognitive abilities, only students who received a 9th grade GPA in Sweden in the years 1998–2001 are included (*n* = 4554 excluded). The final sample comprises 131,864 individuals; 98.8% include cohorts born in 1982–1985, 0.4% is born in 1981 and 0.8% is born in 1986. More than half of the sample, 54.8%, are women. This is because women are overrepresented on the academic tracks in upper secondary schools in Sweden.^1^

To this sample, we match register data from Statistics Sweden (SCB) on several background factors, compulsory and upper secondary school attainment and performance, as well as parents' socioeconomic and migration background. We also match data on municipal school quality indicators from the Swedish National Agency for Education, as well as psychiatric‐related diagnoses and prescriptions from the National Patient Registers and the Prescribed Drug Register administered by the Swedish National Board of Health and Welfare. Given that we exploit individual‐level data on sensitive information such as health and data on children, ethical approval for the study was obtained through the Ethical Review Board in Lund, Sweden (2013/894).

## 5 EMPIRICAL STRATEGY

In this section we describe our empirical strategy; first, we describe how we measure mental ill‐health in our population sample of students; second, we describe how we obtain our school‐level grading bias estimate; and third, we present our empirical model.

### 5.1 Measuring mental ill‐health

Internalizing disorders^2^ and substance use disorders^3^ are the most common psychiatric diagnoses with onset in adolescence and young adulthood in Sweden (The National Board on Health and Welfare, 2020). Our main outcome measure, mental ill‐health, is a dummy indicating the probability of at least one internalizing‐ or substance use disorder diagnosis (inpatient or specialized outpatient care) during the five years following upper secondary school (including graduation year), or at least one prescription for a psychotropic drug^4^ during 2005–2009.^5^ For inpatient diagnosis, both the primary diagnosis as well as the top three secondary diagnoses are included.

The probability of being defined as mentally ill—any internalizing or substance use diagnosis or prescription for a psychotropic drug—is 12.5% in our sample, see Table 1. Prescriptions for psychotropic drugs are more common than internalizing and substance use disorder diagnoses, 11.1% compared to 2.9% and 0.9% respectively. This relates to the fact that diagnoses only capture patients who are diagnosed in inpatient and outpatient care, while prescriptions capture patients who are prescribed drugs from anyone in the healthcare system, including even primary care. Thus, prescriptions for psychotropic drugs is likely a more comprehensive measure of internalizing mental ill‐health than inpatient and outpatient diagnoses.

**TABLE 1:** Descriptive statistics of mental ill‐health

	Total (*N* = 131,864)	Women (*N* = 72,292)	Men (*N* = 59,572)
	Mean	Mean	Mean
Mental ill‐health (any of the below)	0.125	0.156	0.087
Psychotropic prescription	0.111	0.141	0.075
Anxiolytics	0.054	0.069	0.036
Antidepressants	0.077	0.099	0.049
Hypnotics/sedatives	0.041	0.050	0.029
Internalizing disorder diagnosis	0.029	0.038	0.018
Inpatient	0.006	0.009	0.004
Outpatient	0.027	0.035	0.017
Substance use disorder diagnosis	0.009	0.009	0.008
Inpatient	0.004	0.004	0.004
Outpatient	0.005	0.006	0.005

*Note*: This table shows the mean probability of our mental ill‐health outcomes among students graduating from academic track in upper secondary schools in Sweden in the years 2001–2004 (see data section for specific information about the sample). Diagnosis is measured in any of the five years following graduation (including graduation year) and prescription is measured in 2005–2009. Psychotropic drug prescription includes anxiolytics (anti‐anxiety drugs; ATC code N05B), antidepressants (ATC code N06A), and hypnotics/sedatives (sleeping‐pills; ATC code N05C); internalizing disorders diagnosis includes mood (affective) disorders (ICD‐10 codes F30‐39) and neurotic, stress‐related and somatoform disorders (ICD‐10 codes F40‐48), and substance use disorders include mental and behavioral disorders related to psychoactive substance use (ICD‐10 codes F10‐19).

In our sample, the probability of being defined as mentally ill is roughly twice as large among women compared to men. This is true both for inpatient and outpatient diagnosis for internalizing disorders, as well as for prescriptions for anxiolytics, antidepressants and hypnotics/sedatives. The probability of getting diagnosed with substance use disorders is however quite equal between the sexes, inpatient diagnosis for substance use disorder is only slightly more common among men while outpatient diagnosis is slightly more common among women.

**FIGURE 3:**
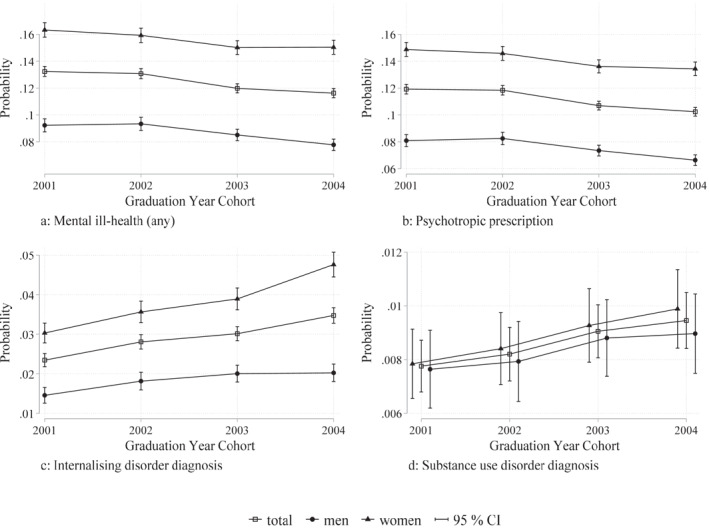
Trends in mental ill‐health. This figure shows the trends in mean probability of our mental ill‐health outcomes among students graduating from academic track in upper secondary schools in Sweden in the years 2001–2004 (see Section 4 for specific information about the sample). Diagnosis is measured in any of the 5 years following graduation (including graduation year) and prescription is measured in 2005–2009. Psychotropic drug prescription includes anxiolytics (anti‐anxiety drugs; ATC code N05B), antidepressants (ATC code N06A), and hypnotics/sedatives (sleeping‐pills; ATC code N05C); internalising disorders diagnosis includes mood (affective) disorders (ICD‐10 codes F30–39) and neurotic, stress‐related and somatoform disorders (ICD‐10 codes F40–48), and substance use disorders include mental and behavioural disorders related to psychoactive substance use (ICD‐10 codes F10–19). In panel d, the markers for men and women are slightly shifted sideways to improve visualisation, but these belong to the same graduation year cohorts.

In Figure 3, the trends in mental ill‐health are shown for women and men in the different graduation year cohorts. Similarly, as for the overall mean probabilities, these trends show that prescriptions for psychotropic drugs and internalizing disorder diagnosis are significantly larger among women compared to men, and while a slight difference in substance use disorder diagnosis emerges over time between women and men, this difference is not statistically significant. The trend of any mental ill‐health decreases over time. This appears to be driven by a significant decrease in prescriptions among women (statistical tests show that the change over time is significant among women but not among men). At the same time, internalizing disorder diagnosis increases significantly among women, and substance use disorder diagnosis increases significantly among women and men. The fact that the probability of mental ill‐health differs between women and men, and between the different mental ill‐health indicators, will be considered in our empirical analyses.

### 5.2 Measuring grading bias

We identify grading bias following Nordin et al. (2019), which in turn is a development of Wikström and Wikström (2005). We measure grading bias as the difference between upper secondary GPA and SweSAT results, on school level, for each cohort.^6^ Some students take the SweSAT more than once. Number of times taking the test (learning effect) and the time before or after graduation (age effect) could impact the test score (Nordin et al., 2019). Therefore, we regress each student *i*'s test score, SweSAT_*i*,*s*,*l*_, on two sets of dummies; the first dummy, *γ*_*i*,*s*_, indicating how many times (*s* = 1,2,…,*s*) the student has taken the test and the second dummy, *δ*_*i*__,__*l*_, indicating the time to graduation *l* when the test is taken:

(1) $${SweSAT}_{i,s,l}=\alpha +\gamma_{i,s}+\delta_{i,l}+{SweSAT}_{i,s,l}^{res}$$

The residual test score, ${SweSAT}_{i,s,l}^{res}$, is saved and a mean residual test score, $\bar{{SweSAT}_{i,t}^{res}}$, is calculated and standardized for each student in graduation year *t*. Next, we regress each student's upper secondary grade in graduation year *t*, GPA_*i*,*t*_, on his or her mean residual test score:

(2) $${GPA}_{i,t}=\bar{{SweSAT}_{i,t}^{res}}+\pi_{i,t}$$

**FIGURE 4:**
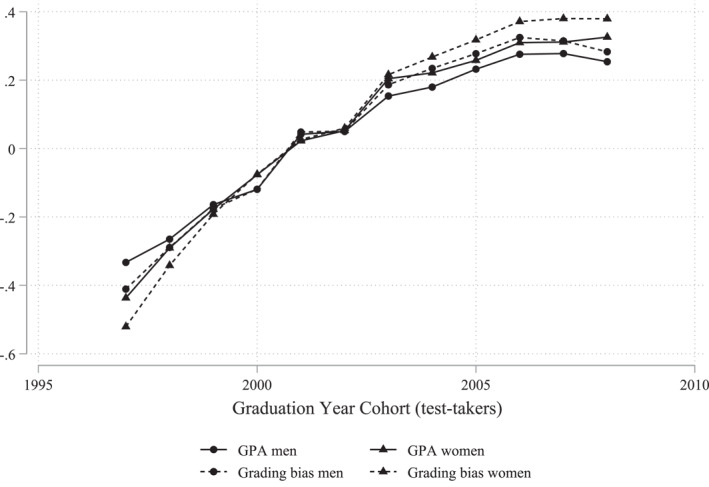
Trends in upper secondary grade point average (GPA) and divergence GPA‐Swedish Scholastic Aptitude Test (SweSAT). This figure shows the GPA (standardized mean) and grading bias (standardized mean difference between GPA and SweSAT) among SweSAT‐taking students graduating from academic track in upper secondary schools in Sweden in the years 1997–2004.

The standardized version of this residual, $\pi_{i,t}$, is the individual level divergence between upper secondary GPA and mean SweSAT. In Figure 4, we show mean upper secondary GPA and mean difference between GPA and SweSAT for the SweSAT‐taking population in each graduation year. The trends show that the increase in mean GPA and grading bias follow each other closely, but that the divergence between GPA and SweSAT increases relatively more than mean GPA, suggesting that grading bias becomes more common during this period. Since we investigate the impact of grading bias over a period when grade inflation is observed in Sweden (Vlachos, 2011; Wikström & Wikström, 2005), we assume that the estimated impact reflects over‐grading.

By averaging the divergence between upper secondary GPA and mean SweSAT on the school level for each school *j*, we get our measure of over‐grading, $\pi_{j,t}$. Since the grading bias measure is calculated on the school level, students who have not yet taken the SweSAT test can also be included. Around half of students in our sample have taken the SweSAT at least once. The share of students that have taken the test decreases gradually from 52% among students graduating in 2001 to 44% among students graduating in 2004. The test‐takers also differ somewhat in terms of background characteristics. Being male, born in Sweden, and having a high educated or a high‐income father all indicate a higher probability of being a test‐taker, as well as having above median compulsory school GPA. The share of test‐takers also varies between the schools, but only around 3% of schools have less than 30% of students taking the test. We consider potential bias from selection in test‐taking in the robustness section.

### 5.3 Our empirical model

We estimate the impact of over‐grading on mental ill‐health in the following linear probability model:

(3) $$Y_{i,j,t}=\alpha_{j}+\delta_{t}+\beta \bar{\pi_{j,t}}+X_{j,t}^{\prime }\gamma +\varepsilon_{i}$$

The school fixed effects, *α*_*j*_, controls for all time‐invariant differences between the schools such as systematic sorting of students into different schools and differences in educational investment quality. We also use graduation year dummies, *δ*_*t*_, to capture cohort variation in mental health. *β* is our coefficient of interest and can be interpreted as the impact of within‐school (*j*) over‐time (*t*) changes in grading bias, *π*_*j,t*_, on the probability of receiving a mental ill‐health‐related diagnosis or prescription, *Y*_*i*,*j*,*t*_. One potential concern with this strategy is that there are some unobserved school‐level input changes that also impact school‐level grades. As mentioned, the grading bias measure is constructed by grades adjusted for a standardized test (the SweSAT), itself not subject to grading bias. SweSAT therefore controls for changing inflow in student cognitive ability, as well as any changes in school level quality that is reflected in the SweSAT results. A vector of control variables is included in X. To account further for changing student characteristics, we also include the mean of 9th grade GPA (assuming this controls for non‐cognitive abilities), the sex balance, the share with any foreign background (foreign‐born or second‐generation with at least one foreign‐born parent), on school‐level for each year. Moreover, if the inflow to different tracks with varying difficulty levels in the same school changes over time, it could have an impact on the grades in that school. Therefore, we control for the proportion of students on each different track for each school and year. A concern may remain that resources may change over time and that this is not adequately controlled for by the SweSAT. To control for such changes, we include several school resources indicators on the municipal level: teacher/pupil ratio, the share of qualified teachers and the share of teachers permanently employed. If resources change between schools, it could also introduce bias. For example, an increased inflow of students with foreign backgrounds would be compensated by increased reimbursements from the municipality. Holmlund et al. (2010) studied how resource allocations changed after the reform and finds that the compensating between schools did not increase over the period. Resources decreased during the time as shown by decreased pupil/teacher ratios, which instead implies that quality, if anything, decreased.

Equation (3) thereby identifies the impact of grading bias on mental health, given that we have adequately controlled for all potential variables correlated with both grading bias and mental health. We assume this to be true, and in support of our assumption we provide several tests in the robustness section below. Standard errors are clustered on the school level in all regressions.

### 5.4 Adjusting grading bias

The unadjusted measure of grading bias $\pi_{j,t}$ is adjusted for each school using the following equation:

(4) $$\bar{\pi_{j,t}}=\alpha_{j}+\delta_{t}+X_{j,t}^{\prime }\gamma +u_{j,t}$$

Saving the residuals $u_{j,t}$ we can then re‐write Equation (3) as:

(5) $$Y_{i,j,t}=\hat{u_{j,t}}+\varepsilon_{i}$$

**FIGURE 5:**
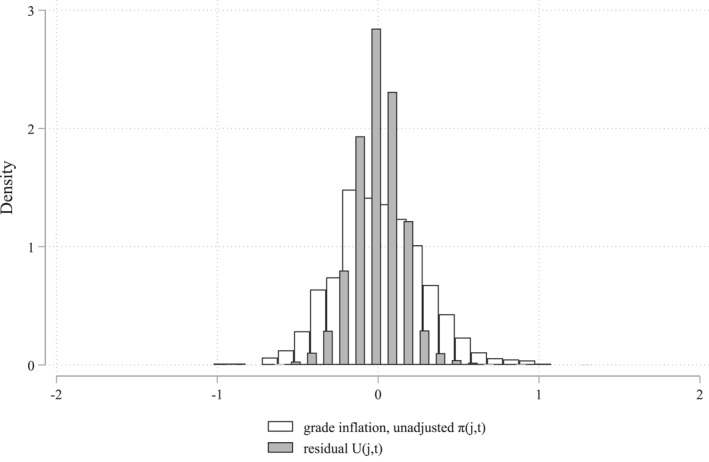
Adjusting the grading bias measure. This figure shows our unadjusted grading bias measure and the residual grading bias measure adjusted for school and graduation year fixed‐effects and additional controls on compulsory grades, tracks, sex and migration share (all on school level for each year), as well as school quality indicators (on municipal level). School‐level grading bias is measured among students graduating from academic track in upper secondary schools in Sweden in the years 2001–2004 (see Section 4 for specific information about the sample).

In Figure 5, to help visualize the variation we are using, we chart our unadjusted measure of grading bias $\bar{\pi_{j,t}}$ and the residuals $\hat{u_{j,t}}$ after adjusting for the included controls in a histogram. This shows that the included controls adjust for some of the variation over time, and when all the controls are included, there is variation left, which we use to study the impact of grading bias on mental health.

## 6 RESULTS

Our main results are presented in Table 2 and show the impact of grading bias on mental ill‐health. Grading bias is negatively associated with our broadest measure of mental ill‐health—any internalizing or substance use diagnosis or prescription for a psychotropic drug—among women, but not in the full sample (women and men together) or among men separately. One standard deviation (sd) increase in grading bias is significantly associated with between 1.9 and 2.3 percentage points (pp) lower probability of mental ill‐health among women, corresponding to relative effects between 11 and 14%. However, only around one percent of schools experienced increases in grading bias of that size, while 39% of schools experienced a quarter of an sd increase in grading bias. Therefore, we interpret our results by corresponding increases in grading bias. A quarter of an sd increase in grading bias is significantly associated with a relative decrease in the probability of mental ill‐health among women just above three percent. The results are quite stable over the different specifications (columns 1–5), but only weakly significant (significant on the 10% level) when tracks are added in specification (3).

**TABLE 2:** Main results: The impact of grading bias on probability of mental ill‐health

	(1)	(2)	(3)	(4)	(5)
Mental ill‐health (any)					
Full sample					
Grading bias	−0.009	−0.004	−0.004	−0.009	−0.010
	(0.006)	(0.006)	(0.006)	(0.005)	(0.006)
Constant	0.125***	0.125***	0.119***	0.063***	0.120**
	(0.000)	(0.000)	(0.009)	(0.012)	(0.059)
Observations	131,841	131,841	131,841	131,841	125,900
Women					
Grading bias	−0.023***	−0.019**	−0.017*	−0.020**	−0.023**
	(0.008)	(0.009)	(0.009)	(0.009)	(0.009)
Constant	0.156***	0.156***	0.168***	0.135***	0.171*
	(0.000)	(0.000)	(0.013)	(0.021)	(0.093)
Observations	72,272	72,272	72,272	72,272	68,927
Men					
Grading bias	−0.002	0.005	0.003	0.002	0.005
	(0.007)	(0.008)	(0.008)	(0.008)	(0.008)
Constant	0.087***	0.087***	0.072***	0.057***	0.125*
	(0.000)	(0.000)	(0.011)	(0.015)	(0.073)
Observations	59,569	59,569	59,569	59,569	56,973

*Note*: This table presents the results for the impact of grading bias on the probability of mental ill‐health among students graduating from academic track in upper secondary schools in Sweden in the years 2001–2004 (see data section for specific information about the sample). The results in each column and for each outcome are from separate regressions. All models include school fixed‐effects and controls for graduation year, additional controls (on school level for each year) are added for each specification with 9th Grade Point Average (GPA) in (2), tracks in (3), sex and migration share in (4) and school quality indicators (on municipal level) in (5). Robust standard errors clustered at the school level are shown in the parentheses.

***p < 0.01, **p < 0.05, *p < 0.1.

The descriptive data show that the probability of mental ill‐health differs substantially between the different mental ill‐health indicators; being prescribed psychotropic drugs is more common than internalizing diagnosis which in turn is substantially more common than substance use disorder diagnosis. We also find that the trends over time go in varying directions for these different measures of mental ill‐health, prescriptions decrease while diagnoses increase. Therefore, we conduct our analyses on the mental ill‐health outcomes—psychotropic drug prescription, internalizing disorder diagnosis and substance use disorder diagnosis—separately. We drop the broad mental ill‐health outcome (any) that contains all of these measures since this is mainly driven by psychotropic prescriptions. Moreover, since the results so far indicate that the impact of grading bias on mental health differs between the sexes, all analyses from now on are performed for men and women separately. We present only our preferred model estimates in Table 3, which includes all the control variables (specification (5) in Table 2) leaving full regression output tables to the Appendix (see Tables A2‐A4).

**TABLE 3:** Main results: The impact of grading bias on probability of psychotropic drug prescription, internalizing‐ and substance use disorder diagnoses

	(1)	(2)	(3)
	Psychotropic drug prescription	Internalizing disorder diagnosis	Substance use disorder diagnosis
Women			
Grading bias	−0.020**	−0.017***	0.001
	(0.009)	(0.006)	(0.003)
Constant	0.125	0.052	0.018
	(0.091)	(0.046)	(0.021)
Observations	68,927	68,927	68,927
Men			
Grading bias	0.005	−0.004	0.002
	(0.007)	(0.004)	(0.003)
Constant	0.108	0.006	−0.002
	(0.068)	(0.035)	(0.022)
Observations	56,973	56,973	56,973

*Note*: This table presents the results for the impact of grading bias on the probability of psychotropic drug prescription, internalizing‐ and substance use disorder diagnoses among students graduating from academic track in upper secondary schools in Sweden in the years 2001–2004 (see data section for specific information about the sample). The results in each column and for each outcome are from separate regressions. All models include school fixed‐effects and the following included controls: graduation year; on school level for each year: 9th grade Grade Point Average (GPA), the share of students on different tracks, sex‐ and migration share; as well as school quality indicators on the municipal level. Robust standard errors clustered at the school level are shown in the parentheses.

***p < 0.01, **p < 0.05, *p < 0.1.

The results show that one sd increase in grading bias is significantly associated with a 2.0 pp lower probability of psychotropic prescription and a 1.7 pp lower probability of internalizing disorder diagnosis, among women. For a quarter of an sd increase in grading bias, this relates to relative effects of four and eight percent lower probability of psychotropic prescription and internalizing disorder diagnosis, respectively, among women. There is no association between grading bias and substance use disorders, neither among men nor women.

### 6.1 Timing

A direct effect of grading bias on mental health would likely occur immediately after the performance feedback received at graduation, while mechanisms through self‐efficacy beliefs or increased schooling likely would show health impacts only after a couple of years or so, depending on how the development of capabilities is affected. To understand these potential underlying mechanisms, it is important to understand when the health impact occurs. Therefore, we investigate how grading bias impacts mental ill‐health in graduation year and the eight following years.

**FIGURE 6:**
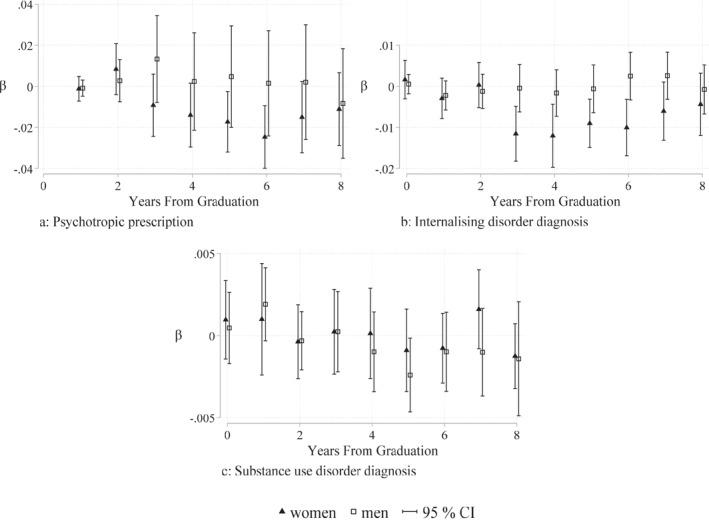
Timing of the impact of grading bias on mental ill‐health. This figure shows the impact of grading bias on our mental ill‐health outcomes (*β* in Equation 3) in graduation year (year 0) and for the eight following years among male and female students graduating from academic track in upper secondary schools in Sweden in the years 2001–2004 (see Section 4 for specific information about the sample). Data on pharmaceutic prescriptions is only available from 2005 which is why we cannot measure this outcome in the year of graduation (year 0). Hence, psychotropic prescription in year 1 is measured in graduation cohort 2004, year 2 is measured in cohorts 2003 and 2004, year 3 in cohorts 2002–2004, and as of year 4 in all the cohorts 2001–2004.

The results are presented in Figure 6 and show that the protective effect of over‐grading on mental health appears among women a couple of years after graduation and is present three to 6 years after graduation, before it diminishes. The trends are similar for psychotropic prescriptions and internalizing disorder diagnosis, but the effect is more persistent and statistically more stable for internalizing diagnosis. Similarly, as in our baseline results, grading bias is not related to substance use disorders among women in any of the years separately. We do however find, among men, indications that over‐grading is related to lower probabilities of substance use disorder starting around 5 years after graduation.

Given that these yearly results indicate that the effect of over grading on mental health is not immediate, but rather appears after a couple of years and is strongest five to 6 years after graduation, we also investigate a more long‐term specification of our dependent variable, probability of diagnosis or prescription in graduation years or any of the 8 years following graduation. These results indicate similar findings to those based on mental ill‐health in the 5 years following graduation. Both definitions show protective effects on both psychotropic prescription and internalizing diagnosis among women, see Table A5 in Appendix, but the long‐term effect on internalizing disorder diagnosis is both stronger and larger compared to the main specification, which is indicated from Figure 6. We find no statistically significant long‐term effect on substance use disorders among men.

### 6.2 Heterogeneity

The human capital development framework of Cunha and Heckman (2007) suggests that background characteristics and skills in the early stages of life are important for the development of capabilities (skills and health). Our main results show that over‐grading has a protective impact on mental health among women but not among men. In this section, we test if grading bias impacts mental health differently in different subgroups of the population stratified by skills (above/below median compulsory school GPA), migration background (foreign‐born or any foreign background), father's education level (higher education or not) and father's income (above/below median income).

The heterogeneity results indicate that the relationship between grading bias and mental ill‐health differs with fathers' SES (see Table 4). The protective relationships between over‐grading and psychotropic drugs and internalizing disorder diagnosis are significant only among women with low SES fathers. Among women with high SES fathers, the estimate sizes for these relationships are substantially smaller, and even reversed for the impact of over‐grading on psychotropic prescriptions among women with high‐income fathers, but the effect estimates are not significantly different from zero. Also, the results indicate that there may be heterogeneity related to fathers' SES among men; over‐grading is related (significant on the 10% level) to a higher probability of prescriptions for psychotropic drugs among men with high‐income and high educated fathers (Table 5).

**TABLE 4:** Heterogeneity analysis: The impact of grading bias on probability of mental ill‐health by socioeconomic background

	(1)	(2)	(3)
	Psychotropic drug prescription	Internalizing disorder diagnosis	Substance use disorder diagnosis
Women			
Grading bias (low educated father)	−0.023**	−0.018***	−0.002
	(0.011)	(0.007)	(0.003)
Grading bias (high educated father)	−0.010	−0.010	0.008
	(0.017)	(0.011)	(0.005)
Constant	0.126	0.050	0.003
Observations: 68,925	(0.091)	(0.045)	(0.004)
Grading bias (low‐income father)	−0.046***	−0.020**	−0.001
	(0.012)	(0.008)	(0.004)
Grading bias (high‐income father)	0.014	−0.012	0.003
	(0.014)	(0.009)	(0.004)
Constant	0.111	0.056	0.015
Observations: 68,925	(0.090)	(0.046)	(0.021)
Men			
Grading bias (low educated father)	−0.005	−0.009	−0.000
	(0.009)	(0.005)	(0.002)
Grading bias (high educated father)	0.025*	0.005	0.007
	(0.013)	(0.007)	(0.004)
Constant	0.128*	0.003	−0.003
Observations: 56,971	(0.068)	(0.035)	(0.022)
Grading bias (low‐income father)	−0.009	−0.010	0.004
	(0.011)	(0.007)	(0.004)
Grading bias (high‐income father)	0.019*	0.002	−0.000
	(0.010)	(0.005)	(0.003)
Constant	0.114*	0.008	−0.003
Observations: 56,972	(0.069)	(0.036)	(0.022)

*Note*: This table presents heterogeneity by socioeconomic background for the impact of grading bias on the probability of psychotropic drug prescription, internalizing‐ and substance use disorder diagnoses among students graduating from academic track in upper secondary schools in Sweden in the years 2001–2004 (see data section for specific information about the sample). The results in each column and for each outcome are from separate regressions in each subgroup. All models include school fixed‐effects and the following included controls: graduation year; on school level for each year: 9th grade Grade Point Average (GPA), the share of students on different tracks, sex‐ and migration share; as well as school quality indicators on the municipal level. Robust standard errors clustered at the school level are shown in the parentheses.

***p < 0.01, **p < 0.05, *p < 0.1.

**TABLE 5:** Heterogeneity analysis: The impact of grading bias on probability of mental ill‐health by migration background

	(1)	(2)	(3)
	Psychotropic drug prescription	Internalizing disorder diagnosis	Substance use disorder diagnosis
Women			
Grading bias (born in Sweden)	−0.022**	−0.017***	0.000
	(0.009)	(0.006)	(0.003)
Grading bias (foreign‐born)	0.004	−0.013	0.010
	(0.028)	(0.019)	(0.009)
Constant	0.132	0.056	0.016
Observations: 68,921	(0.090)	(0.046)	(0.021)
Grading bias (Swedish background)	−0.026**	−0.023***	−0.002
	(0.010)	(0.007)	(0.003)
Grading bias (foreign background)	0.001	0.003	0.009
	(0.019)	(0.012)	(0.006)
Constant	0.137	0.058	0.019
Observations: 68,923	(0.091)	(0.047)	(0.021)
Men			
Grading bias (born in Sweden)	0.004	−0.003	0.004
	(0.007)	(0.004)	(0.003)
Grading bias (foreign‐born)	0.016	−0.004	−0.010
	(0.026)	(0.013)	(0.007)
Constant	0.102	0.010	−0.004
Observations: 56,962	(0.070)	(0.035)	(0.022)
Grading bias (Swedish background)	0.007	−0.003	0.006*
	(0.009)	(0.005)	(0.003)
Grading bias (foreign background)	−0.001	−0.005	−0.007*
	(0.014)	(0.007)	(0.004)
Constant	0.114	0.006	−0.001
Observations: 56,971	(0.070)	(0.036)	(0.022)

*Note*: This table presents heterogeneity by migration background for the impact of grading bias on the probability of psychotropic drug prescription, internalizing‐ and substance use disorder diagnoses among students graduating from academic track in upper secondary schools in Sweden in the years 2001–2004 (see data section for specific information about the sample). The results in each column and for each outcome are from separate regressions in each subgroup. All models include school fixed‐effects and the following included controls: graduation year; on school level for each year: 9th grade Grade Point Average (GPA), the share of students on different tracks, sex‐ and migration share; as well as school quality indicators on the municipal level. Robust standard errors clustered at the school level are shown in the parentheses.

***p < 0.01, **p < 0.05, *p < 0.1.

The heterogeneity results indicate differences based on migration background as well. The protective relationships between over‐grading and psychotropic drugs and internalizing disorder diagnosis are significant only among women who are born in Sweden and among women with no foreign background. Interestingly, while our main results show no relationship between grading bias and substance use disorders, the heterogeneity results indicate that there may be a link between over‐grading and substance use disorders among men, but that this relationship differs with migration background. Over‐grading is related (significant on the 10% level) to a lower probability of substance use disorders among men with foreign backgrounds and a higher probability of these disorders among Swedish‐background men.

Finally, there is heterogeneity also based on skills. The protective relationship between over‐grading and internalizing disorder diagnosis is significant only among women who had above median GPA in compulsory school (see Table 6), for below‐median GPA women the impact is substantially smaller but not significantly different from zero. The effect estimates for the relationship between over‐grading and psychotropic prescription that we find in the full group of women is not significant in either subgroup when stratified by compulsory school GPA, but they are of similar size.

**TABLE 6:** Heterogeneity analysis: The impact of grading bias on probability of mental ill‐health by compulsory school GPA

	(1)	(2)	(3)
	Psychotropic drug prescription	Internalizing disorder diagnosis	Substance use disorder diagnosis
Women			
Grading bias (below‐median GPA)	−0.017	−0.011	−0.001
	(0.013)	(0.007)	(0.005)
Grading bias (above‐median GPA)	−0.022	−0.024***	0.005
	(0.014)	(0.008)	(0.003)
Constant	0.147	0.054	0.016
Observations: 68,924	(0.092)	(0.047)	(0.021)
Men			
Grading bias (below‐median GPA)	−0.004	−0.006	0.002
	(0.010)	(0.005)	(0.004)
Grading bias (above‐median GPA)	0.016	−0.001	0.003
	(0.012)	(0.007)	(0.003)
Constant	0.123*	0.004	−0.001
Observations: 56,970	(0.069)	(0.036)	(0.022)

*Note*: This table presents heterogeneity by compulsory school GPA for the impact of grading bias on the probability of psychotropic drug prescription, internalizing‐ and substance use disorder diagnoses among students graduating from academic track in upper secondary schools in Sweden in the years 2001–2004 (see data section for specific information about the sample). The results in each column and for each outcome are from separate regressions in each subgroup. All models include school fixed‐effects and the following included controls: graduation year; on school level for each year: 9th grade Grade Point Average (GPA), the share of students on different tracks, sex‐ and migration share; as well as school quality indicators on the municipal level. Robust standard errors clustered at the school level are shown in the parentheses.

***p < 0.01, **p < 0.05, *p < 0.1.

### 6.3 Cause‐specific results

In our main analyses, the diagnosis probability is measured by combined inpatient and outpatient diagnoses. Moreover, the probability of prescriptions for psychotropic drugs is measured by anxiolytics, antidepressants, and hypnotics/sedatives (sleeping pills) combined, preparations that are mainly used to treat internalizing disorders such as anxiety and depression. Likely though, inpatient and outpatient diagnoses reflect somewhat different manifestations of mental problems, especially regarding symptom severity. Thus, the impact of grading bias may differ between inpatient and outpatient diagnoses, and between the different drugs.

In Table 7, we investigate the impact of grading bias separately across inpatient and outpatient diagnoses and find that over‐grading is associated with a 1.4 pp lower probability of outpatient diagnoses among women. For inpatient diagnoses, the relationship is only weakly significant. Our cause‐specific results moreover show the protective impact of over‐grading on prescriptions for psychotropic drugs among women is driven by antidepressants. Over‐grading is associated with a 2.0 pp lower probability of prescription for antidepressants among women. We also find weak indications that over‐grading is associated with a 0.8 pp higher probability of prescriptions for anxiolytics among men. Similar to our baseline results, grading bias is not related to in‐ or outpatient substance use disorder diagnoses (results not shown).

**TABLE 7:** Cause‐specific analyses: The impact of grading bias on inpatient‐/outpatient internalizing disorder diagnoses and different psychotropic drugs

	(1)	(2)	(3)	(4)	(5)
	Inpatient internalizing disorder diagnosis	Outpatient internalizing disorder diagnosis	Anxiolytics	Anti‐depressants	Hypnotics/Sedatives
Women					
Grading bias	−0.005*	−0.014***	−0.006	−0.020**	0.000
	(0.003)	(0.005)	(0.006)	(0.008)	(0.006)
Constant	0.000	0.049	0.153**	0.011	0.112*
	(0.027)	(0.044)	(0.064)	(0.080)	(0.058)
Observations: 68,927					
Men					
Grading bias	−0.003	−0.004	0.008*	0.000	−0.003
	(0.002)	(0.004)	(0.005)	(0.006)	(0.005)
Constant	−0.010	0.010	0.073	0.082	0.012
	(0.018)	(0.033)	(0.052)	(0.053)	(0.043)
Observations: 56,973					

*Note*: This table presents the results for the impact of grading bias on the probability of in‐ and outpatient internalizing disorder diagnoses and different psychotropic drugs among students graduating from academic track in upper secondary schools in Sweden in the years 2001–2004 (see data section for specific information about the sample). The results in each column and for each outcome are from separate regressions. All models include school fixed‐effects and the following included controls: graduation year; on school level for each year: 9th grade Grade Point Average (GPA), the share of students on different tracks, sex‐ and migration share; as well as school quality indicators on the municipal level. Robust standard errors clustered at the school level are shown in the parentheses.

***p < 0.01, **p < 0.05, *p < 0.1.

## 7 ROBUSTNESS

Our identification strategy is to isolate the effect of grading bias by studying within‐school over‐time changes in the divergence between mean GPA and SweSAT results, controlling for compulsory school grades, student composition and school production changes. In this section, we provide a number of tests to investigate the robustness of this strategy. First, we assess the balance of our sample with respect to grading bias using conditional independence tests; second, we investigate the impact of placebo‐grading bias on mental health during a period when we know there was no grade inflation; third, we investigate if selection in taking the SweSAT is likely to bias our results; and fourth, we assess the sensitivity of the results to functional form.

### 7.1 Conditional independence tests

Our key identifying assumption is that conditional on the included control variables, grading bias is independent of mental health. To test the validity of this assumption we investigate if the measure for grading bias (*π*_*j*,*t*_) is correlated to predetermined characteristics among the students. We do this by including several predetermined characteristics that may also be linked to mental health.—father's income and education, migration background and below‐median 9th grade GPA—as dependent variables in Equation (3). If the sample is balanced in terms of these observed pre‐determined characteristics, it is more likely to be balanced also in terms of unobserved characteristics, and we can identify the true impact of grading bias on mental health. The results show that grading bias, in general, is not related to these characteristics, see Table A6 in the Appendix. When only school and graduation year fixed effects are used as controls (column (1) of Table A6), grading bias is positively associated with fathers' years of schooling and associated with having below median GPA in 9th grade. However, the inclusion of compulsory school grades yields estimates very close to zero with no substantial effect on standard errors. Further additional controls for tracks, sex, migration share, and school quality indicators have no additional effect (see columns 3, 4 and 5 respectively), which implies that additional controls are not necessary. The conditional independence tests moreover reveal a weak association between grading bias and foreign background (see columns 4–6) which should be noted, but in general, these tests supports our assumption of conditional independence.

To further test this assumption, we also control our main results for linear time trends at the municipality level, which are shown to have negligible impact on our results, see Tables A1–A4 (column 6) in the Appendix.

### 7.2 Placebo test

Our identification strategy relies on the assumption that the discrepancy between final grades and SweSAT results reflect over‐grading and that what our results reveal is actually an effect of this school‐level over‐grading on mental health. We observe that the general grade inflation in our sample decreased significantly in 2003 (Figure 1), although the shares of students with the highest grades kept increasing more modestly for another couple of years (Figure 2). One concern is that something else, besides over‐grading, is affecting the mental health among students graduating from upper secondary schools in Sweden. Therefore, to further test the robustness of our results we perform so called placebo tests. This is done by running our main model on a sample of students exposed to significantly lower levels, or even no level, of over‐grading, namely the cohorts graduating in years 2005–2008. These tests show placebo estimates that are close to zero, see Table A7 in the Appendix, indicating no effect on mental health, which supports our identification strategy and indicates that our results are not reflecting some spurious relationship between grading bias and mental health.

### 7.3 Selection in test‐taking

One potential source of bias is if there is selection in taking the SweSAT. Selection between schools or based on background characteristics should not be a problem since we measure within‐school over‐time effects of over‐grading, and we control for changing composition of students. However, if the share of students who take the test changes within schools over time, and this is related to some factor that is unobserved, it could introduce bias. We test if selection in test‐taking is a problem by including an indicator for the share of test‐taking in each school and year. Additionally, we include the interaction (linear and quadratic) between the test‐taking indicator and compulsory school grades and find that share of test‐takers is not related to our outcome measures, nor does the inclusion of this variable and the interactions have any impact on our results, see Table A8 in Appendix.

### 7.4 Sensitivity to functional form

**FIGURE 7:**
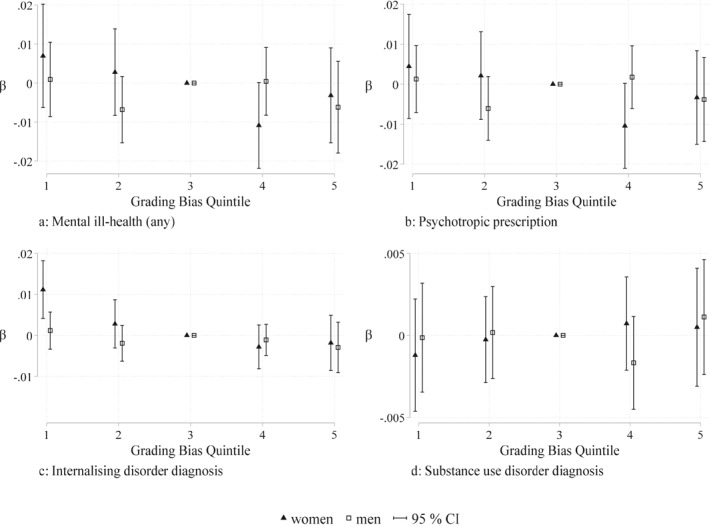
Sensitivity analysis of functional form: The impact of grading bias in quintiles on mental ill‐health. This figure presents the impact of grading bias in quintiles on the probability of psychotropic drug prescription, internalising‐ and substance use disorder diagnoses among students graduating from academic track in upper secondary schools in Sweden in the years 2001–2004 (see Section 4 for specific information about the sample). Grading bias is divided into quintiles 1–5 from lowest bias (Q1) to highest bias (Q5), Q3 is the reference group and represents the quintile of around median levels of bias. All models include school fixed‐effects and the following included controls: graduation year; on school level for each year: ninth grade Grade Point Average (GPA), the share of students on different tracks, sex‐ and migration share; as well as school quality indicators on the municipal level. Standard errors are clustered at the school level.

Our baseline model assumes a linear relationship between grading bias and mental ill‐health. We assess the linear functional form assumption of our model between grading bias and mental ill‐health by estimating the relationship between grading bias and mental ill‐health in quintiles of grading bias from low bias level (Q1) to high bias level (Q5), see Figure 7. Compared to the reference group (Q3, reflecting close to median levels of grading bias) psychotropic prescription is lower among women in the slightly higher bias quintile (Q4), but the difference is only significant on 10% level. For internalizing diagnosis, it is only women in the lowest bias quintile (Q1) that differs from Q3 with a significantly higher probability of diagnosis. These results indicate that the relationship between grading bias and mental ill‐health might not be completely linear, however, the direction of the sign for the *β* estimates in the different quintiles indicate that the protective effect we find between grading bias and psychotropic prescription and internalizing disorder diagnosis in our main results is present for women in schools with higher bias levels, that is, where we know over‐grading took place.

## 8 DISCUSSION

Our results show that women who were exposed to over‐grading in Sweden were substantially less likely to suffer from mental ill‐health in the following years. Being exposed to a quarter of an sd increase in grading bias, a level of over‐grading that is observed in more than a third of the schools, is associated with an eight percent lower probability of being diagnosed for internalizing disorders such as depression and anxiety, and a four percent lower probability of being prescribed mental ill‐health related drugs such as antidepressants and anxiolytics. We find no effects of grading errors among men. Results using a non‐linear function of grading bias suggest that the findings are driven by the upper half of grading bias, that is by higher levels of over grading. We show that, conditional on our included control variables, the measure we use to estimate grading bias appears independent to several predetermined characteristics such as father's SES, compulsory school grades and being foreign‐born. We do find a weak relationship between grading bias and having any foreign background, which should be noted as a potential threat to the conditional independence of grading bias. However, given the stability of our results through several robustness checks, for example, placebo tests, and that the results are robust to the inclusion of important control variables and even municipality level time trends, indicates that our independent variable of interest is balanced, and provides support that our model is, in fact, a valid model to investigate grading bias effects.

We hypothesized that over‐grading impacts mental health, either through a direct effect of changes in feedback on mental health or through a self‐efficacy effect by which over‐grading increase self‐confidence, motivation and performance and thereby impact mental health. We also hypothesize that over‐grading impacts mental health through increased higher education opportunities which are correlated to better mental health. Our results on the timing of the protective effect on mental health among women find that this protective effect occurs three to 4 years after graduation, and the effect is strong for a couple of years before it reduces somewhat. This indicates that there is not a direct effect of grading bias on mental health. Indirect effects through higher education enrollment and later labor market outcomes would likely impact mental health later in life. We have investigated this potential mechanism by studying the effect of grading bias on higher education enrollment, any higher education, and years of higher education, and find that only the latter is significantly impacted by over‐grading, see Table A9 in Appendix.^7^ Over‐grading is significantly associated with more years of schooling both among women (0.157 years) and men (0.176 years), which indicates that it is less likely that the grading bias effect on mental health among women is driven only by higher education enrollment. Additional years of schooling is a potential mechanism for the protective effect of over‐grading, and instead supports the hypothesis that self‐efficacy may be a relevant mechanism in the protective relationship between over‐grading and mental health among women.

Our finding of clear differences across sexes raises the question if women and men are differently exposed to over‐grading. Previous work by Lindahl (2007) suggests that girls are over‐graded in comparison to boys in Sweden. She compares grades between girls and boys who have the same score on national tests in different subjects and finds that girls receive higher final grades in these subjects at the end of compulsory school. Possibly, teachers include other criteria besides the national test score in the final grade and if girls outperform boys in these tasks, it could be an explanation of this result. In another study from Sweden, Hinnerich et al. (2011) uses both blind and non‐blind examples of the same test to compare grading between boys and girls, and show that boys are not discriminated against in grading. Our findings suggest that grades and grading biases increased both among men and women during our study period (see Figures 1, 2, 3), suggesting trends of inflated grades and exposure to over‐grading in both groups. However, grades and grading biases increased relatively more among young women, so it is possible that over‐grading was more intense among women, which would explain that there is a difference, but the question is if it is likely that the exposure varies to the extent that it would impact mental health among women quite substantially, while no effect at all among men. Women are overrepresented in mental ill‐health, especially for internalizing disorders, which is another potential explanation for the difference between sexes, if levels of mental ill‐health already are low among men over‐grading may be less likely to have an impact. Yet, we find strong and significant effects for two independent indicators of mental ill‐health among women, psychotropic prescription and internalizing diagnosis, even though internalizing diagnosis among women is substantially less common than psychotropic prescription, both among women and men. This indicates that it is not only differences in the probability of mental ill‐health between women and men that drives the results. A third possibility is that the mental ill‐health outcomes differ between women and men. While the outcomes are measured exactly the same, previous research indicates that men and women seek mental healthcare to a varying extent, and at different symptom levels, in which case the level of severity of the mental illness may differ between diagnosed or drug‐prescribed women and men (Kovess‐Masfety et al., 2014). If so, this could potentially explain the different effects.

Besides heterogeneity between women and men, we also find that the impact of over‐grading on mental health appears to differ based on socioeconomic and migration background, and somewhat also by skills. Over‐grading is protective against mental ill‐health mainly among women with low‐SES fathers and among women with no foreign background. Given that there is a documented socioeconomic gradient in mental health (see e.g., Linder et al. (2020)), this heterogeneity would actually reduce mental health gaps, all other things equal. There are also some (weaker) indications that over‐grading increases the risk for psychotropic drug prescriptions among men with higher education and high‐income fathers, and that over‐grading increases the risk of substance use disorders among men with no foreign background and reduces the risk of substance use disorders among men with foreign backgrounds. This is interesting because in the main analyses we do not find any association between over‐grading and any of our mental ill‐health outcomes in the full sample of men. Intuitively it seems reasonable that the admission distortion resulting in competition with higher ability peers could induce more stress and anxiety‐related disorders if the father is high educated, if the expectations on performing well then also are higher. But it is not as straightforward as to why foreign background alters the relationship between over‐grading and substance use diagnosis. Substance use is in general lower among adolescents with foreign backgrounds (Hjern & Allebeck, 2004), but could the mechanisms through self‐efficacy or university enrollment be stronger among young men with foreign backgrounds? Other studies have also investigated heterogeneity in grading bias consequences on school performance. For example (Lavy & Sand, 2015), finds that over‐grading of boys in primary school had positive impacts on later school achievements among boys with high educated parents and among boys with European or North‐American backgrounds, and that this re‐ranking between the sexes had negative impacts on achievements among girls with low‐educated parents and among girls with Asian or African background. Socioeconomic and migration background thus appears to be important factors to consider in the consequences of grading bias.

In general, grading bias and especially grade inflation is seen as something bad. Systemic grading bias creates welfare costs due to an inefficient allocation of investments in higher education and, on an individual level, grading bias creates unfair labor market opportunities between students. Our findings show that the consequences of systematic grading bias are complex and go beyond the schooling and labor market consequences previously suggested in the literature—over‐grading even impacts mental health—which means that grades themselves, independent of knowledge changes, are important for both health and skill formation among students, and therefore impacts life chances. This means that policies that directly or indirectly impact grading in schools may also impact health and life chances among young individuals. For example, prior to the 90's school reforms in Sweden, the norm‐referenced relative grading system was to some extent compensating; grades were often normally distributed within a class (The Swedish National Agency for Education, 2005), which meant that high‐performing students were under‐graded and low‐performing students were over‐graded. Recent reports have also shown that grading in Sweden still to some extent reflect the general level of performance at schools, that grading is more restrictive in schools with many high‐performing students and conversely less restrictive in schools with many low‐performing students (The Swedish National Agency for Education, 2019). Since there is a well‐documented socioeconomic gradient in mental health these examples of grading bias would likely reduce these gaps, all other things equal. This has implications for highly relevant policy questions, not only in Sweden but also internationally where the debate regarding, for example, normalized or goal‐oriented reference systems, blind/non‐blind grading of tests, and the choice between standardized or discretionary grading systems, is frequent. There is a shift toward an increased use of standardized grading systems (Lundahl et al., 2016). For example, in China, India, Israel and Japan the systems are now exclusively standardized (Diamond & Persson, 2016), but a mix of standardized and discretionary systems are more common (Lundahl et al., 2016).

Similarly, there is a male/female gradient in mental health. The probabilities of internalizing diagnosis and psychotropic prescription were twice as high among women than men in our sample, 14.1% versus 7.5% for psychotropic drug prescription and 3.8% versus 1.8% for internalizing disorder diagnosis, see Table 1. The protective effect of a sd increase in grading bias on psychotropic prescription (2.0 pp) and internalizing diagnosis (1.7 pp) among women corresponds to between 30 and 85% of the gaps in these outcomes. Even a quarter of a sd increase in grading bias, that we know was exhibited by more than a third of schools, relates to substantial parts of these gaps, 8 and 21% respectively, implying that grading bias had unintended effects on reducing gender gaps in mental health. Our results also relate to how we understand and respond to events and shocks. Recent findings show that mental ill‐health is rising among the young and the pandemic appears to have exacerbated this trend (Thorisdottir et al., 2021). At the same time, we are facing a situation where distance schooling during the pandemic appears to have induced grade inflation (Karadag, 2021). While these trends individually are of concern, our results imply that the protective effects of over‐grading may mitigate some of the challenges students are facing due to the pandemic.

We identify grading bias as the school‐level divergence between mean GPA and SweSAT in Sweden 2001–2004 when grade inflation was observed (Vlachos, 2011; Wikström & Wikström, 2005). We can also observe that both grades and grading bias increase in our sample, and the bias increases relatively more than grades, which is why we assume that what we measure is over‐grading. However, one potential shortcoming with this strategy is that we cannot say anything about the absolute levels of grading bias. We show that the relationship differs with level of bias and that the protective impact on mental ill‐health is driven by median‐to higher‐bias levels, where we assume that students are exposed to over‐grading. Another potential disadvantage is that we are not able to fully investigate mechanisms through increased higher education enrollment and advantageous labor market outcomes. If grading bias would affect mental health later in life through these mechanisms, they are not captured by the sample period of our analysis. The longer‐term health consequences of over‐grading are left to future research.

## 9 CONCLUSION

We show that over‐grading, receiving a higher grade not reflecting one's true knowledge or performance, has a non‐negligible protective impact on mental health among women. Our findings contribute to a more comprehensive picture of the consequences of grading bias and grade inflation impacts. Grading bias impacts health and skills formation in the years following graduation, but the consequences differ based on sex, socioeconomic and migration background, which means that grading bias has the potential to impact health inequality.

## CONFLICT OF INTEREST

The authors declare that there is no conflict of interest.

## Supporting information

Supporting Information S1

## Data Availability

The data that support the findings of this study are available from Statistics Sweden and the Swedish National Board on Health and Welfare. Restrictions apply to the availability of these data, which were used under license for this study. Data are available from Statistics Sweden and the Swedish National Board on Health and Welfare with the permission of these parties.

## ENDNOTES

^1^
The sex balance is more even if the sample is not restricted to academic track‐students, 50.6 percent of students graduating from vocational and academic tracks between 2001–2004 are women.

^2^
Internalizing disorders include mood (affective) disorders (ICD‐10 codes F30‐39) and neurotic, stress‐related and somatoform disorders (ICD‐10 codes F40‐48). In general, these are emotional or behavioral mental problems that are internalized—kept to themselves—by the individual, often characterized by a change in mood or affect, for example, depression, a neurotic reaction to an environmental or stressful situation, or a general manifestation of anxiety.

^3^
Substance use disorders include mental and behavioral disorders related to psychoactive substance use (ICD‐10 codes F10‐19), diagnoses that are attributable to acute intoxication, harmful use, dependence syndromes and other conditions related to the use of one or more psychoactive substances such as alcohol, narcotics or tobacco.

^4^
Psychotropic drugs are commonly used to treat internalizing disorders and include anxiolytics (anti‐anxiety drugs; ATC code N05B), hypnotics/sedatives (sleeping‐pills; ATC code N05C) and antidepressants (ATC code N06A).

^5^
The reason we use a different time‐period to measure prescriptions is that register data on prescriptions was not available prior to 2005. Given that the follow up period is the same for all cohorts, if prescriptions vary with age, it is possible that the probability for prescription varies between the cohorts. However, this is controlled for by graduation year dummies.

^6^
In Nordin et al. (2019) the grading bias measure was averaged up separately for women and men with the motivation that grading bias might differ between the sexes. In this study, we have calculated the grading bias measure on school level for women and men together, but we have checked the robustness of our results to this alternative identification of grading bias and there is no substantial difference in our results or conclusions.

^7^
Nordin et al. (2019) finds that over‐grading increases higher education enrollment among men only, and higher education achievements among men and women.
