# Supplementary material for: Grading bias and young adult mental health
Source: Health Econ. 2022 Dec 7;32(3):675–96. doi: 10.1002/hec.4639 (PMC10108256; doi:10.1002/hec.4639)
Supplement: Supplementary file 1 — Supporting Information S1 [file HEC-32-675-s001.docx]

## Appendix

**Table A1:**

**The Impact of Grading Bias on Probability of Mental Ill-Health (any)**

**Full specification, including time trends**

|  | (1) | (2) | (3) | (4) | (5) | (6) |
| --- | --- | --- | --- | --- | --- | --- |
| **Mental ill-health (any)** | | | | | | |
| **Full sample** | | | | | | |
| Grading bias | -0.009 | -0.004 | -0.004 | -0.009 | -0.010 | -0.010* |
|  | (0.006) | (0.006) | (0.006) | (0.006) | (0.006) | (0.006) |
| Constant | 0.125*** | 0.125*** | 0.119*** | 0.0630*** | 0.120** | 0.126** |
|  | (0.000) | (0.000) | (0.009) | (0.012) | (0.059) | (0.059) |
| *Observations* | *131,841* | *131,841* | *131,841* | *131,841* | *125,900* | *125,900* |
| **Women** | | | | | | |
| Grading bias | -0.023*** | -0.019** | -0.017* | -0.020** | -0.023** | -0.023** |
|  | (0.008) | (0.009) | (0.009) | (0.009) | (0.009) | (0.009) |
| Constant | 0.156*** | 0.156*** | 0.168*** | 0.135*** | 0.171* | 0.185** |
|  | (0.000) | (0.000) | (0.013) | (0.020) | (0.093) | (0.094) |
| *Observations* | *72,272* | *72,272* | *72,272* | *72,272* | *68,927* | *68,927* |
| **Men** | | | | | | |
| Grading bias | -0.002 | 0.005 | 0.003 | 0.002 | 0.005 | 0.005 |
|  | (0.007) | (0.008) | (0.008) | (0.008) | (0.008) | (0.008) |
| Constant | 0.087*** | 0.087*** | 0.072*** | 0.057*** | 0.125* | 0.121 |
|  | (0.000) | (0.000) | (0.011) | (0.015) | (0.073) | (0.073) |
| *Observations* | *59,569* | *59,569* | *59,569* | *59,569* | *56,973* | *56,973* |

Note: This table represents Table 2 in the main text including a column controlling for linear time trends. The results in each column and for each outcome are from separate regressions. All models include school fixed-effects and controls for graduation year, additional controls (on school level for each year) are added for each specification with 9^th^ Grade Point Average (GPA) in (2), tracks in (3), sex and migration share in (4), and (on municipal level) school quality indicators in (5) and linear time trends in (6). Robust standard errors clustered at the school level are shown in the parentheses. ***p<0.01, **p<0.05, *p<0.1

**Table A2:**

**The Impact of Grading Bias on Probability of Psychotropic Prescription**

**Full specification, including time trends**

|  | (1) | (2) | (3) | (4) | (5) | (6) |
| --- | --- | --- | --- | --- | --- | --- |
| **Psychotropic prescription** | | | | | | |
| **Full sample** | | | | | | |
| Grading bias | -0.006 | -0.002 | -0.002 | -0.007 | -0.008 | -0.008 |
|  | (0.006) | (0.006) | (0.006) | (0.006) | (0.006) | (0.006) |
| Constant | 0.112*** | 0.111*** | 0.103*** | 0.051*** | 0.086 | 0.090 |
|  | (0.000) | (0. 000) | (0.008) | (0.011) | (0.057) | (0.058) |
| *Observations* | *131,841* | *131,841* | *131,841* | *131,841* | *125,900* | *125,900* |
| **Women** | | | | | | |
| Grading bias | -0.020** | -0.016* | -0.014 | -0.017** | -0.020** | -0.019** |
|  | (0.008) | (0.008) | (0.008) | (0.008) | (0.009) | (0.009) |
| Constant | 0.142*** | 0.142*** | 0.146*** | 0.113*** | 0.125 | 0.131 |
|  | (0.000) | (0.000) | (0.013) | (0.020) | (0.091) | (0.091) |
| *Observations* | *72,272* | *72,272* | *72,272* | *72,272* | *68,927* | *68,927* |
| **Men** | | | | | | |
| Grading bias | 0.000 | 0.006 | 0.005 | 0.004 | 0.005 | 0.005 |
|  | (0.007) | (0.007) | (0.007) | (0.007) | (0.007) | (0.007) |
| Constant | 0.075*** | 0.076*** | 0.063*** | 0.052*** | 0.108 | 0.105 |
|  | (0.000) | (0.000) | (0.010) | (0.015) | (0.068) | (0.069) |
| *Observations* | *59,569* | *59,569* | *59,569* | *59,569* | *56,973* | *56,973* |

Note: This table shows the full regression output for Table 3 column (1) in the main text. The results in each column and for each outcome are from separate regressions. All models include school fixed-effects and controls for graduation year, additional controls (on school level for each year) are added for each specification with 9^th^ Grade Point Average (GPA) in (2), tracks in (3), sex and migration share in (4), and (on municipal level) school quality indicators in (5) and linear time trends in (6). Robust standard errors clustered at the school level are shown in the parentheses. ***p<0.01, **p<0.05, *p<0.1

**Table A3:**

**The Impact of Grading Bias on Probability of Internalising Disorder Diagnosis**

**Full specification, including time trends**

|  | (1) | (2) | (3) | (4) | (5) | (6) |
| --- | --- | --- | --- | --- | --- | --- |
| **Internalising disorder diagnosis** | | | | | | |
| **Full sample** | | | | | | |
| Grading bias | -0.009*** | -0.009*** | -0.009** | -0.010*** | -0.011*** | -0.010*** |
|  | (0.003) | (0.003) | (0.003) | (0.003) | (0.004) | (0.004) |
| Constant | 0.029*** | 0.029*** | 0.023*** | 0.007 | 0.022 | 0.027 |
|  | (0.000) | (0.000) | (0.004) | (0.006) | (0.030) | (0.030) |
| *Observations* | *131,841* | *131,841* | *131,841* | *131,841* | *125,900* | *125,900* |
| **Women** | | | | | | |
| Grading bias | -0.016*** | -0.016*** | -0.014*** | -0.015*** | -0.017*** | -0.017*** |
|  | (0.005) | (0.005) | (0.005) | (0.005) | (0.006) | (0.006) |
| Constant | 0.039*** | 0.039*** | 0.034*** | 0.025** | 0.052 | 0.064 |
|  | (0.000) | (0.000) | (0.006) | (0.010) | (0.046) | (0.046) |
| *Observations* | *72,272* | *72,272* | *72,272* | *72,272* | *68,927* | *68,927* |
| **Men** | | | | | | |
| Grading bias | -0.004 | -0.003 | -0.004 | -0.004 | -0.004 | -0.003 |
|  | (0.004) | (0.004) | (0.004) | (0.004) | (0.004) | (0.004) |
| Constant | 0.018*** | 0.018*** | 0.014*** | 0.009 | 0.006 | 0.002 |
|  | (0.000) | (0.000) | (0.005) | (0.007) | (0.035) | (0.036) |
| *Observations* | *59,569* | *59,569* | *59,569* | *59,569* | *56,973* | *56,973* |

Note: This table shows the full regression output for Table 3 column (2) in the main text. The results in each column and for each outcome are from separate regressions. All models include school fixed-effects and controls for graduation year, additional controls (on school level for each year) are added for each specification with 9^th^ Grade Point Average (GPA) in (2), tracks in (3), sex and migration share in (4), and (on municipal level) school quality indicators in (5) and linear time trends in (6). Robust standard errors clustered at the school level are shown in the parentheses. ***p<0.01, **p<0.05, *p<0.1

**Table A4:**

**The Impact of Grading Bias on Probability of Substance Use Disorder Diagnoses**

**Full specification, including time trends**

|  | (1) | (2) | (3) | (4) | (5) | (6) |
| --- | --- | --- | --- | --- | --- | --- |
| **Substance use disorder diagnosis** | | | | | | |
| **Full sample** | | | | | | |
| Grading bias | 0.000 | 0.001 | 0.001 | 0.001 | 0.002 | 0.001 |
|  | (0.002) | (0.002) | (0.002) | (0.002) | (0.002) | (0.002) |
| Constant | 0.009*** | 0.009*** | 0.006** | 0.007** | 0.010 | 0.010 |
|  | (0.000) | (0.000) | (0.002) | (0.003) | (0.014) | (0.014) |
| *Observations* | *131,841* | *131,841* | *131,841* | *131,841* | *125,900* | *125,900* |
| **Women** | | | | | | |
| Grading bias | 0.000 | 0.001 | 0.001 | 0.001 | 0.001 | 0.001 |
|  | (0.002) | (0.003) | (0.003) | (0.003) | (0.003) | (0.003) |
| Constant | 0.009*** | 0.009*** | 0.007** | 0.012** | 0.018 | 0.022 |
|  | (0.000) | (0.000) | (0.003) | (0.005) | (0.021) | (0.021) |
| *Observations* | *72,272* | *72,272* | *72,272* | *72,272* | *68,927* | *68,927* |
| **Men** | | | | | | |
| Grading bias | -0.000 | 0.001 | 0.001 | 0.001 | 0.002 | 0.002 |
|  | (0.002) | (0.002) | (0.003) | (0.003) | (0.003) | (0.003) |
| Constant | 0.008*** | 0.008*** | 0.003 | 0.001 | -0.002 | -0.003 |
|  | (0.000) | (0.000) | (0.003) | (0.005) | (0.022) | (0.022) |
| *Observations* | *59,569* | *59,569* | *59,569* | *59,569* | *56,973* | *56,973* |

Note: This table shows the full regression output for Table 3 column (3) in the main text. The results in each column and for each outcome are from separate regressions. All models include school fixed-effects and controls for graduation year, additional controls (on school level for each year) are added for each specification with 9^th^ Grade Point Average (GPA) in (2), tracks in (3), sex and migration share in (4), and (on municipal level) school quality indicators in (5) and linear time trends in (6). Robust standard errors clustered at the school level are shown in the parentheses. ***p<0.01, **p<0.05, *p<0.1

**Table A5:**

**The Impact of Grading Bias on Long-term Probability of Mental Ill-Health (all outcomes, up to 9 years following graduation)**

|  | (1) | (2) | (3) | (4) |
| --- | --- | --- | --- | --- |
|  | **Mental ill-health (any)** | **Psychotropic drug prescription** | **Internalising disorder diagnosis** | **Substance use disorder diagnosis** |
| **Women** | | | | |
| Grading bias | -0.030*** | -0.020** | -0.026*** | -0.002 |
|  | (0.010) | (0.009) | (0.007) | (0.003) |
| Constant | 0.153 | 0.127 | 0.049 | 0.015 |
| *Observations: 68,927* | (0.098) | (0.094) | (0.064) | (0.023) |
| **Men** | | | | |
| Grading bias | 0.007 | 0.006 | 0.000 | 0.000 |
|  | (0.009) | (0.009) | (0.006) | (0.003) |
| Constant | 0.139* | 0.160* | 0.033 | 0.035 |
| *Observations: 56,973* | (0.078) | (0.094) | (0.053) | (0.029) |

Note: This table presents the results for the impact of grading bias on the probability of mental ill-health (any), psychotropic drug prescription, internalising- and substance use disorder diagnoses among women and men graduating from academic track in upper secondary schools in Sweden in the years 2001 – 2004 (see data section for specific information about the sample). The outcome reflects the probability of diagnosis or prescription in graduation year or the eight years following graduation. The results in each column and for each outcome are from separate regressions. All models include school fixed-effects and controls for graduation year; on school level for each year: 9^th^ grade Grade Point Average (GPA), the share of students on different tracks, sex- and migration share; as well as school quality indicators on the municipal level. Robust standard errors clustered at the school level are shown in the parentheses. ***p<0.01, **p<0.05, *p<0.1

**Table A6 Conditional Independence Tests:**

**The Impact of Grading Bias on Predetermined Characteristics**

|  | (1) | (2) | (3) | (4) | (5) |
| --- | --- | --- | --- | --- | --- |
| **Fathers’ education** | | | | | |
| Grading bias | 0.171** | 0.008 | 0.017 | 0.087 | 0.075 |
|  | (0.079) | (0.083) | (0.085) | (0.074) | (0.076) |
| Constant | 11.560*** | 11.560*** | 11.740*** | 12.520*** | 12.460*** |
|  | (0.001) | (0.001) | (0.110) | (0.119) | (0.737) |
| **Fathers’ income** | | | | | |
| Grading bias | 0.040 | -0.016 | -0.022 | 0.014 | 0.016 |
|  | (0.034) | (0.035) | (0.034) | (0.028) | (0.029) |
| Constant | 6.952*** | 6.953*** | 6.956*** | 7.358*** | 7.229*** |
|  | (0.000) | (0.000) | (0.045) | (0.049) | (0.224) |
| **Foreign-born** | | | | | |
| Grading bias | -0.002 | 0.006 | 0.009 | 0.007 | 0.007 |
|  | (0.006) | (0.006) | (0.006) | (0.006) | (0.006) |
| Constant | 0.074*** | 0.074*** | 0.068*** | 0.046*** | 0.100* |
|  | (0.000) | (0.000) | (0.007) | (0.011) | (0.051) |
| **Foreign background** | | | | | |
| Grading bias | -0.002 | 0.012 | 0.018* | 0.015* | 0.017* |
|  | (0.009) | (0.009) | (0.009) | (0.009) | (0.009) |
| Constant | 0.207*** | 0.207*** | 0.197*** | 0.167*** | 0.183** |
|  | (0.000) | (0.000) | (0.013) | (0.017) | (0.083) |
| **Below median 9^th^ grade Grade Point Average (GPA)** | | | | | |
| Grading bias | -0.107*** | 0.007 | 0.003 | -0.003 | -0.003 |
|  | (0.018) | (0.006) | (0.006) | (0.006) | (0.006) |
| Constant | 0.501*** | 0.500*** | 0.497*** | 0.430*** | 0.373*** |
|  | (0.000) | (0.000) | (0.008) | (0.012) | (0.058) |
| *Observations* | *131,841* | *131,841* | *131,841* | *131,841* | *125,900* |

Note: This table presents the results for the impact of grading bias for selected predetermined characteristics among students graduating from academic track in upper secondary schools in Sweden in the years 2001 – 2004 (see data section for specific information about the sample). The results in each column and for each outcome are from separate regressions. All models include school fixed-effects and controls for graduation year and an indicator for missing outcome variable, additional controls (on school level for each year) are added for each specification with 9^th^ grade GPA in (2), tracks in (3), sex and migration share (not for “Foreign-born” and “Foreign background”) in (4) and school quality indicators (on municipal level) in (5). Robust standard errors clustered at the school level are shown in the parentheses. ***p<0.01, **p<0.05, *p<0.1

**Table A7 Placebo-test:**

**The Impact of Placebo-Grading Bias on Mental Ill-Health (all outcomes)**

|  | (1) | (2) | (3) | (4) |
| --- | --- | --- | --- | --- |
|  | **Mental ill-health (any)** | **Psychotropic**  **drug prescription** | **Internalising disorder diagnosis** | **Substance use disorder diagnosis** |
| **Women** | | | | |
| Grading bias | 0.006 | 0.003 | -0.001 | -0.001 |
|  | (0.009) | (0.008) | (0.006) | (0.003) |
| Constant | -0.001 | 0.042 | -0.042 | -0.020 |
| *Observations: 70,206* | (0.062) | (0.048) | (0.041) | (0.025) |
| **Men** | | | | |
| Grading bias | 0.007 | 0.001 | 0.006 | 0.003 |
|  | (0.006) | (0.006) | (0.004) | (0.003) |
| Constant | -0.014 | 0.017 | -0.007 | -0.003 |
| *Observations: 62,994* | (0.043) | (0.037) | (0.033) | (0.024) |

Note: This table presents the results for the impact of exposure to no grading bias, or low levels of grading bias, on the probability of mental ill-health (any), psychotropic drug prescription, internalising- and substance use disorder diagnoses among women and men graduating from academic track in upper secondary schools in Sweden in the years 2005 – 2008. The results in each column and for each outcome are from separate regressions. All models include school fixed-effects and controls for graduation year; on school level for each year: 9^th^ grade Grade Point Average (GPA), the share of students on different tracks, sex- and migration share; as well as school quality indicators on the municipal level. Robust standard errors clustered at the school level are shown in the parentheses. ***p<0.01, **p<0.05, *p<0.1

**Table A8 Sensitivity Analysis:**

**The Impact of Grading Bias on Probability of Mental Ill-Health (all outcomes)**

**Controlling for selection in test-taking**

|  | (1) | (2) | (3) | (4) |
| --- | --- | --- | --- | --- |
|  | **Mental ill-health (any)** | **Psychotropic drug prescription** | **Internalising disorder diagnosis** | **Substance use disorder diagnosis** |
| **Women** | | | | |
| Grading bias | -0.025*** | -0.021** | -0.017*** | 0.001 |
|  | (0.009) | (0.009) | (0.005) | (0.003) |
| SweSAT_j,t_ | 0.135 | -0.011 | 0.068 | 0.072* |
|  | (0.128) | (0.118) | (0.075) | (0.042) |
| SweSAT_j,t_*GPA^9th^ | 0.064 | 0.061 | 0.013 | 0.015 |
|  | (0.061) | (0.058) | (0.037) | (0.018) |
| SweSAT_j,t_^2 | -0.078 | 0.068 | -0.065 | -0.062 |
|  | (0.129) | (0.116) | (0.073) | (0.041) |
| SweSAT_j,t_^2*GPA^9th^^2 | -0.136* | -0.130* | -0.056 | -0.012 |
|  | (0.072) | (0.069) | (0.038) | (0.017) |
| Constant | 0.120 | 0.116 | 0.032 | -0.006 |
| *Observations: 68,927* | (0.097) | (0.095) | (0.050) | (0.027) |
| **Men** | | | | |
| Grading bias | 0.005 | 0.005 | -0.004 | 0.002 |
|  | (0.008) | (0.007) | (0.004) | (0.003) |
| SweSAT_j,t_ | -0.069 | -0.079 | -0.034 | -0.002 |
|  | (0.127) | (0.118) | (0.055) | (0.035) |
| SweSAT_j,t_*GPA^9th^ | -0.034 | -0.037 | 0.009 | -0.011 |
|  | (0.069) | (0.061) | (0.031) | (0.020) |
| SweSAT_j,t_^2 | 0.083 | 0.103 | 0.040 | -0.012 |
|  | (0.124) | (0.117) | (0.052) | (0.034) |
| SweSAT_j,t_^2*GPA^9th^^2 | -0.041 | -0.037 | -0.060 | 0.010 |
|  | (0.079) | (0.070) | (0.037) | (0.025) |
| Constant | 0.143* | 0.128* | 0.014 | 0.001 |
| *Observations: 56,973* | (0.079) | (0.073) | (0.038) | (0.024) |

Note: This table presents the results for the impact of grading bias on the probability of mental ill-health (any), psychotropic drug prescription, internalising- and substance use disorder diagnoses among women and men graduating from academic track in upper secondary schools in Sweden in the years 2001 – 2004 (see data section for specific information about the sample). The results in each column and for each outcome are from separate regressions. All models include school fixed-effects and controls for graduation year; on school level for each year: 9^th^ grade Grade Point Average (GPA), the share of students on different tracks, sex- and migration share; school quality indicators on the municipal level; share of SweSAT-takers in each school and year, as well as the interaction between SweSAT-share and compulsory school GPA. Robust standard errors clustered at the school level are shown in the parentheses. ***p<0.01, **p<0.05, *p<0.1

**Table A9:**

**The Impact of Grading Bias on Higher Education**

|  |  | (1) | (2) | (3) |
| --- | --- | --- | --- | --- |
|  |  | **Higher education enrolment** | **Any higher education** | **Years of higher education** |
| **Women** | | | | |
| Grading bias (above-median GPA) |  | -0.003 | 0.000 | 0.157*** |
|  |  | (0.012) | (0.013) | (0.057) |
| Constant |  | 0.882*** | 0.851*** | 2.772*** |
| *Observations: 68,927* |  | (0.105) | (0.110) | (0.493) |
| **Men** |  |  |  |  |
| Grading bias (above-median GPA) |  | 0.017 | 0.019 | 0.176*** |
|  |  | (0.015) | (0.015) | (0.059) |
| Constant |  | 0.817*** | 0.735*** | 2.178*** |
| *Observations: 56,973* |  | (0.125) | (0.137) | (0.594) |

Note: This table presents the results for the impact of grading bias on the probability of higher education enrolment, any higher education, and years of higher education among women and men graduating from academic track in upper secondary schools in Sweden in the years 2001 – 2004 (see data section for specific information about the sample). The results in each column and for each outcome are from separate regressions. All models include school fixed-effects and controls for graduation year; on school level for each year: 9^th^ grade Grade Point Average (GPA), the share of students on different tracks, sex- and migration share; as well as school quality indicators on the municipal level. Robust standard errors clustered at the school level are shown in the parentheses. ***p<0.01, **p<0.05, *p<0.1
